# Association of *GSTTI*, *M1* and Polymorphism in *GSTPI* with Chronic Periodontal Disease in a Pakistani Population

**DOI:** 10.3390/genes14020455

**Published:** 2023-02-10

**Authors:** Kainat Arshad, Uzma Ishfaq, Muhammad Asif, Atif Akbar, Kehkashan Fatima Pitafi, Muhammad Rehan Mulghani, Uzman Shaheen, Suleman Saeed, Muhammad Arif, Ahsan Bashir, Muhammad Farooq, Alan Henry Brook, Furhan Iqbal

**Affiliations:** 1 Department of Zoology, Ghazi University, Dera Ghazi Khan 32200, Pakistan; 2 Institute of Zoology, Bahauddin Zakariya University, Multan 60800, Pakistan; 3 Institute of Molecular Biology and Biotechnology, Bahauddin Zakariya University, Multan 60800, Pakistan; 4 Department of Statistics, Bahauddin Zakariya University, Multan 60800, Pakistan; 5 Senior Registrar Medicine, Teaching Hospital, Dera Ghazi Khan 32200, Pakistan; 6 Pathology Department, Dera Ghazi Khan Medical College, Dera Ghazi Khan 32200, Pakistan; 7 Dental School, University of Adelaide, Adelaide 5005, Australia

**Keywords:** chronic periodontitis, *GSTT1*, *GSTM1*, rs1695, *GSTP1*

## Abstract

Objective: Chronic periodontal disease (CP) is a multifactorial infectious and inflammatory disease that occurs due to the challenge between the immune response of the host and specific periodontal bacteria, and that can lead to tooth loss due to damage inflicted to the supporting tissue. The current study investigates the genotypes of the *GSTM1* and *GSTT1* genes, along with the allelic frequency of the single nucleotide polymorphism [SNP; rs1695] in the GSTP1 gene and correlates them individually or in various combinations with the incidence of CP. Methods: A total of 203 clinically confirmed CP patients and 201 control subjects were enrolled from Multan and Dera Ghazi Khan Districts in Pakistan from April to July 2022. Multiplex Polymerase Chain Reaction (PCR) and tetra-primer amplification refractory mutation system–polymerase chain reaction (T-ARMS–PCR) approaches were applied to determine the genotypes of the studied GSTs. The association of rs1695 in *GSTP1* with CP was studied both individually and in various combinations with *GSTM1* and *T1*. Results: The absence of *GSTM1*, the presence of *GSTT1* and the presence of the mutant allele (G) at rs1695 in *GSTP1* were found to be significantly associated with CP. Patients aged between 10 and 30 years were more affected by CP. Conclusion: Our results indicate that the genotypes of the analyzed GSTs affect the levels of protection from oxidative stress and may therefore influence the disease progression in CP.

## 1. Introduction

Chronic Periodontal disease is among the most important oral diseases contributing to the burden of chronic diseases around the globe, and is a major public health problem [1]. Periodontal disease is a general term that represents a spectrum of inflammatory diseases affecting the periodontium—the oral connective tissue consisting of four structures that support the teeth: the cementum, gingiva, alveolar bone, and the periodontal ligament [2,3]. Each of the components of the periodontium varies in their cellular composition, degree of metabolic activity, mineralization, and disease susceptibility [4]. During the World Workshop on the classification of Periodontal and Peri-implant diseases and conditions, held in 2017, periodontal disease and conditions were classified into three major categories, each with subcategories: 1. Periodontal health and gingival diseases covering periodontal and gingival health, gingivitis caused by bacteria and gingivitis not caused by biofilm; 2. Periodontitis including necrotizing diseases, periodontitis as a manifestation of systemic disease and periodontitis; 3. Other conditions affecting the periodontium including systemic diseases, periodontal/endodontic lesions, mucogingival deformities, etc. [5,6].

Periodontitis is known to affect a large number of populations around the world, including both developed and underdeveloped countries [1]. The documented prevalence of periodontitis in various parts of Pakistan has been variable, and, according to a recent report, the prevalence of this disease was 37% in Punjab, 40% in Sindh, 20% in Khyber Pakhtunkhwa and 3% in Baluchistan [7]. Periodontitis is strongly associated with factors such as low socio-economic status and poor access to healthcare services [8]. The high prevalence of this disease in Pakistan is also attributed to poor oral hygiene and a lower level of public awareness regarding oral hygiene [9].

Mucosal tissues of the oral epithelium are exposed to several environmental stressors, including periodontal bacteria and tobacco that promote inflammatory states [10]. The host produces and releases inflammatory mediators to counter the oxidative stress caused by these microorganisms and chemicals. Polymorphonuclear leucocytes are activated during CP and exhibit increased production of reactive oxygen species (ROS) that are associated with periodontal tissue destruction and osteoclastic bone resorption [11]. As aerobic organisms, ROS production is inevitable in humans, and humans have an antioxidant defense system consisting of enzymes and non-enzymatic materials to cope with these cations and anions [12]. Glutathione-S-transferases (GST) are enzymes that play a key role in the detoxification of such substances and prevent them from damaging important cellular components [13]. Human cytosolic GSTs are highly polymorphic and consist of seven classes: Alpha, Mu, Pi, Theta, Sigma, Omega and Zeta [14]. Among these, the most extensively studied genes are Mu (μ), Pi (π) and Theta (θ) as the polymorphisms in these genes have been linked to a number of inflammatory disorders [15].

GSTs produced during chronic inflammation can be used as biomarkers for CP. In the study conducted by Varghese et al. [13], a decrease in GST levels in both the gingival tissue and gingival crevicular fluid was observed in CP patients when compared with the control group. Polymorphisms in GST genes are associated with a number of diseases including CP [13,16,17]. Genetic polymorphisms are disease modifiers in periodontitis, affecting its severity and clinical course, while susceptibility to disease development varies from person to person [18]. The relevance of different gene polymorphisms with CP is still under debate. Therefore, the present study was designed to report the association of presence or absence of the *GSTM1* and *GSTT1* genes, as well as rs1695 in the *GSTP1* gene, with CP in a sample of the Pakistani population.

## 2. Materials and Methods

### 2.1. Ethical Approval

Experimental protocols were reviewed and approved by the Ethical Committee of Department of Zoology, Ghazi University Dera Ghazi Khan (Pakistan) (Application Number: GU/Zool. Ethics/22-09).

### 2.2. Patients and Sample Collection

A total of 203 clinically confirmed chronic periodontitis patients from the District Head Quarter Hospital (DHQ) Dera Ghazi Khan were included in the study conducted between April and July 2022, after receiving an informed consent from each patient. The required sample size was calculated by using the formula N = (Z^2^P (1 − P) × D)/e^2^ following Jones et al. [19], where Z is the standard normal variate, P is proportion, D is the design effect and e is the level of precision. The required sample size from this calculation was 397, in keeping with the 18% prevalence of CP in the study area. The sample consisted of 105 males and 98 females, with ages ranging from 10 to 70 years. The age and sex distributions matched those of the control group (N = 201), the subjects of which were from the same ethnic group in Dera Ghazi Khan District, but who were not suffering from CP. The control group included 105 males and 96 females with their ages ranging from 11 to 69 years. The patients were examined by calibrated dental surgeons and the parameters considered for the clinical diagnosis and inclusion of cases included the width of the attached gingiva, the Plaque Index, the number of teeth in each quadrant and probing the pocket depth. Clinical and radiographic signs of moderate (3–4 mm) and severe (5 mm or more) attachment loss were recorded. Patients excluded from the sample included pregnant or lactating females, those with rampant caries, those having oral or extraoral piercing in or around the oral cavity, those requiring antibiotic coverage for dental treatment and patients who had received periodontal treatment within the last 6 months.

A blood sample (3–5 mL) was collected from each subject from their median cubital vein in a blood collection tube containing 0.5 M Ethylene Diamine Tetra Acetic Acid (EDTA) as an anticoagulant. A questionnaire was completed for each enrolled subject to gather the epidemiological and clinical data associated with periodontal status (orthodontic treatment, pocket probing depth (PPD), bleeding on probing (BOP), clinical attachment level (CAL), Plaque Index and the number of teeth in each quadrant).

### 2.3. DNA Extraction

Deoxyribonucleic acid (DNA) was extracted from the whole blood using a DNA extraction protocol described previously [20].

### 2.4. Amplification and Genotyping of GSTT1 and GSTM1

A multiplex PCR was carried out to determine the presence or absence of *GSTT1* and *GSTM1* in the subjects, following Jamil et al. [20]. Cytochrome P450, family 1, subfamily A, polypeptide 1 (*CYP1A1*) gene (exon 7) was also amplified as an internal control. The oligonucleotide primers used for the amplification of these genes are presented in Table S1 (Supplementary Materials). A 50 μL master mixture was prepared for PCR containing 5 μL of template DNA, 5 μL of PCR buffer, 3.5 μL of MgCl_2_ (25 mM), 2 μL of each primer (12 Pm), 2 μL of dNTPs (2 mM) and 1 μL of DNA polymerase (Thermo Fisher Scientific, Waltham, MA, USA). The thermal profile for *GSTM1*, *GSTT1* and *CYP1A1* amplification consisted of an initial denaturation for 5 min at 94 °C, followed by 30 cycles of a denaturation for 2 min at 94 °C, an annealing step for 1 min at 59 °C, elongation for 1 min at 72 °C and a final extension for 10 min at 72 °C [20].

### 2.5. Tetra-Primer ARMS–PCR-Based Amplification of rs1695 in GSTP1

A tetra-primer amplification refractory mutation system–PCR (T-ARMS–PCR) was performed on genotype Ileu 105 Val (rs1695 A/G) in exon 5 of the *GSTP1* gene as reported by Jamil et al. [20].

The primers used in this T-ARMS–PCR are shown in Table S1. A total of 25 μL of reaction mixture was prepared comprising 3 μL of template DNA, 5 μL of PCR buffer, 2 μL of MgCl_2_ (25 mM), 2 μL of each primer (12 Pm), 2 μL of dNTPs (2 mM) and 1 μL of Taq DNA polymerase (Thermo Fisher Scientific, Waltham, MA, USA). Additionally, rs1695 in GSTP1 was amplified by applying the following thermal conditions: initial denaturation for 5 min at 95 °C, followed by 35 cycles of denaturation for 30 s at 95 °C, annealing for 30 s at 62 °C, elongation for 30 s at 72 °C and the final extension for 10 min at 72 °C [20].

### 2.6. Statistical Analysis

Statistical Package Minitab (version 19, State College, PA, USA) was used for data analysis. Significance level was set at *p* ≤ 0.05. The chi-square test was applied to compare the genotype, allelic frequency, and epidemiological factors between the case and control groups. Hardy–Weinberg Equilibrium was calculated for rs1695 in the GSTP1 gene to estimate the genetic diversity among the studied population.

## 3. Results

### 3.1. Presence or Absence of GSTM1 and GSTT1 and Their Association with Chronic Periodontitis

During the multiplex PCR, oligonucleotide primers generated amplicons of 215 base pairs (bp) and 480 bp when the *GSTM1* and *GSTT1* genes were present, respectively. These bands were absent in the *GSTM1* and *GSTT1* gene null subjects, whereas the amplification of the *CYP1A1* gene always resulted in 312 bp DNA fragments in all subjects being used as a positive control.

When the distribution of *GSTM1* was compared between the enrolled subjects, the chi-square test results revealed that the *GSTM1* genotype varied significantly between the CP patients and controls (*p* < 0.001). The absence of *GSTM1* (null genotype) was associated with the incidence of CP (Table 1). The *GSTT1* genotype similarly varied significantly between the CP patients and controls (*p* < 0.001), with the presence of the *GSTT1* being associated with CP (Table 1).

### 3.2. Genotypic and Allelic Frequency of rs1695 in GSTP1 and Their Association with Chronic Periodontitis 

When the Tetra-primer Amplification Refractory Mutation System–polymerase chain reaction (T-ARMS–PCR) was applied to the rs1695 Single Nucleotide Polymorphism (SNP) in the *GSTP1* gene outer primers, an amplicon of 467 base pairs was generated that was visible for all enrolled subjects. While the primers specific for the homozygous wild (AA) genotype and the homozygous mutant (GG) genotype amplified products of 233 and 290 bp, respectively, the amplification of samples from patients who had the heterozygous genotype (AG) at rs1695 resulted in amplicons of both sizes (233 and 290 bp).

When the genotype at *rs1695* in the *GSTP1* gene was examined among controls, it was observed that the wild type (AA) was the most common genotype (88%), followed by the heterozygous (AG) (10%) and the homozygous mutants (GG) (2%). The pattern observed in CP patients was AA (57%), AG (29%), and GG (14%). The genotype frequency of the heterozygous and homozygous mutants was significantly higher in the CP patients than in controls (*p* < 0.001)) (Table 2). 

When the allelic frequency at *rs1695* in the *GSTP1* gene was compared among controls, it was observed that “A” (Wild type) allele (93%) was more frequent than the “G” (Mutant) allele (7%) (Table 2). The pattern in the CP patients was A (71%), G (29%) (Table 2). The allelic frequency varied significantly (*p* ˂ 0.001) between the healthy controls and the CP patients, with the controls more frequently having the wild allele (A) and the CP patients more often having the mutant allele (G), which indicated that the mutant allele was associated with the incidence of CP (Table 2).

We calculated the observed (HO) and expected heterozygosity (HE), as well as the *p* value, for the Hardy–Weinberg equilibrium for both the case and control groups. Analysis of our results indicated significant deviations from the Hardy–Weinberg Law for both the control (x^2^ = 10.8409; *p* < 0.01) and case (x^2^ = 17.7542; *p* < 0.01) subjects, indicating genetic diversity in both the studied groups (Table S1).

### 3.3. GSTs Interactions and Their Association with Chronic Periodontitis

Analysis of genotype frequencies indicated that certain genotypes in combination with two or more GSTs significantly increased the risk of developing chronic periodontal disease. All the genotype combinations that had a significantly higher frequency within the control group were protecting combinations, while those that had a higher frequency in the patient group were associated with a higher risk of developing CP (Table 3). Patients were more susceptible to chronic periodontitis if: both the *GSTMI* and *T1* were absent (*p* < 0.001); they were *GSTM1* null and had any genotype at *GSTP1* (*p* < 0.001); they were *GSTT1* null with heterozygous (AG) and mutant (GG) genotype (*p* < 0.001); or they had *GSTT1* present but *GSTM1* absent with any genotype at rs1695 in *GSTP1* (*p* < 0.001) (Table 3).

### 3.4. Association of Demographic Factors with the Incidence of Chronic Periodontal Disease

When demographic factors were compared between the CP and control group patients, the chi-square test results revealed that age (*p* < 0.001) was the only factor that was significantly associated with the incidence of CP. Patients in the age range of 10–30 years were more susceptible to chronic periodontal disease. Neither gender nor smoking habit was significantly associated with CP (Table 4).

## 4. Discussion

The enhanced production of ROS, or their decreased clearance through the antioxidant activities, results in lipid peroxidation within the cells, leading to several pathological conditions including CP [21]. The role of ROS and antioxidants in CP has been investigated, and a number of natural and synthetic materials may have the potential to reduce gingival inflammation and improve periodontal parameters [13,22]. 

The effect of different genetic polymorphisms in CP is still being investigated. In the present study, the absence of the *GSTM1* and the presence of the *GSTT1 genes have been shown to be significantly associated* with CP (Table 1). GST null genotypes are associated with decreased enzymatic activity and poor antioxidant protection that promote oxidative stress resulting in the development of periodontitis [23]. Our results reflect those of Sánchez et al. [15], who reported that the absence of the *GSTM1* and *the* presence of the *GSTT1 genes* increase the susceptibility to dental caries in a Mexican population. Similarly, Ortega et al. [17] analyzed the presence or absence of the *GSTM1 and GSTT1* genes, along with polymorphisms in the *GSTP1* gene, in a Mexican population and reported significant differences in genotypes between periodontitis case and control groups. Their patients with chronic periodontitis had a higher frequency of null and mutant polymorphisms in the *GSTM1*, *GSTT1* and *GSTP1* genes as compared with the control Mexican population and it was concluded that the presence of these polymorphisms may be a risk factor for the development of CP. Our results are also in agreement with Concolino et al. [16]. These authors reported a higher frequency of the *GSTM1* null genotype in Caucasian patients from central Italy, who was suffering from CP as compared with a control group. Results of these studies are contradictory to the observations of Kim et al. [24], who did not find any association between the *GSTM1* null genotype and CP in Korean population. In a recent study from Serbia, Jakovljevic et al. [25] reported that the *GSTM1* and *GSTT1* null genotypes were separately, as well as concomitantly, associated with an increased risk for apical periodontitis development.

There are very limited data available in the literature showing the prevalence of rs1695 in the *GSTP1* gene in relation to CP. Hence, the data generated in this investigation provide a baseline for future studies. In the present investigation, the heterozygous (AG) and the mutant alleles (GG) at rs1695 in the *GSTP1* gene were associated with CP as their frequency was significantly higher in the CP patients than in controls (Table 2). Our results agree with Sánchez et al. [15], who reported that heterozygous and mutant alleles at rs1695 in the *GSTP1* gene were associated with susceptibility to dental caries in the Mexican population.

The analysis of Hardy–Weinberg equilibrium is used to estimate the heterozygosity value and to calculate the genetic diversity level of a population [26]. During the present study, the Hardy–Weinberg equilibrium was found to be disturbed in both the case and the control groups, and the observed and expected heterozygosity of the case and control subjects differed significantly from each other. High heterozygosity observed during the present study indicates high genetic diversity among the enrolled CP cases and the healthy controls (Table S1). Several factors are known to disturb the Hardy–Weinberg equilibrium including mutations, natural selection, nonrandom mating, genetic drift, as well as gene flow [27], and these factors are also applicable to the population that was enrolled in the present study.

Results from studies of individual SNPs are inconsistent as each individual SNP alters the function of a single gene among the many that are involved in the progression of CP. However, disease progression can be due to the interaction of several proteins. A single SNP usually has a modest effect, but the same biological events are more greatly affected by different SNPs in different genes and how these SNP combinations interact. We performed SNP–SNP interaction analysis and examined various SNP combinations from the GSTs screened in the present study to identify the combination of SNPs that are most likely to be associated with the risk of developing CP. The absence of the GSTM1 gene and the presence of the GSTT1 gene were found to be risk factors for the onset of CP when these genes were analyzed individually (Table 1), while the GSTs combination analysis revealed a number of genotype combinations that were found to be associated with CP (Table 3). 

These observations support our hypothesis that it is the combination of genotypes with one or more SNPs that plays an important role in health and disease. Our results indicated that the genotype at rs1695 in the *GSTP1* gene plays an important role in the CP development, regardless of the genotypes of the *GSTM1* and *GSTT1* genes; it was the heterozygous (AG) and mutant (GG) genotype at rs1695 of the *GSTP1* gene that was always linked to CP, making it an interesting candidate-SNP to be considered for analysis in CP-related studies in various populations (Table 3).

The analysis of the risk factors included in the present study revealed that subjects in the age range of 10–30 years were more susceptible to CP, while gender and smoking habit had no significant association with CP (Table 4). Our observations are complementary to the World Health Organization’s report [28] which states that approximately 15 to 20% of the middle-aged adult population (aged 30 years or more) is affected by CP. Rajasekar et al. [29] also reported that subjects from India, within the age range of 18–30 years, had a higher incidence of CP. Our results are also in agreement with Shehzad et al. [9] as they reported in their recent investigation—carried out in the Khyber Pakhtunkhwa Province of Pakistan—that despite the high prevalence of periodontitis among the enrolled subjects, the disease was not limited to a particular gender. Contrary to these observations, some studies have reported a higher prevalence of CP in male subjects than in females [29,30]. Our results are contradictory to Ortega et al. [17] as they had reported that smoking was associated with the incidence of CP in a Mexican population. On the other hand, Küchler et al. [18] found no significant differences in the mean age and gender when compared between periodontitis patients and healthy controls enrolled in Serbia.

The number of samples is a limitation of the present study as the greater the sample size, the higher is the power of the observations and obtained results. The number of genes and SNPs related to CP in our study was also limited: many other genes and their SNPs are reported to be associated with CP in various ethnic groups around the globe. There is a need to explore more genes and SNPs related to CP in order to accurately assess the susceptibility (arising from these genes) to this disease in the Pakistani population. However, the present study is valuable, as it is the first study from Pakistan that reports the genotype at three GSTs with reference to CP.

## 5. Conclusions

In conclusion, the prevalence of CP in Pakistan is high and this serious dental issue must be addressed to improve the oral health-related quality of life in the local population. We are reporting that GST genotypes play a significant role in the progression of CP as we have observed that the *GSTM1* nulls, as well as heterozygous and mutants at rs1695 in the *GSTP1* gene may confer increased susceptibility to CP in the local population. Subjects aged from 10 to 30 years were more susceptible to CP, but this does not mean that 10-year-old children will have an increased predisposition to periodontitis, as the translation from a genetic predisposition to a clinical manifestation may take some years. Further large-scale studies should be undertaken to establish the role of *GSTs* in the onset of CP.

## Figures and Tables

**Table 1:** Distribution of the presence or absence of the *GSTM1* and *GSTT1* genes among CP cases and control subjects enrolled in the present study. The *p*-value represents the outcome of the chi-square test calculated for each combination.

Genotype	Controls	Case	Chi-Square Value	*p*-Value
*GSTM1* Present	185	66	152.1	<0.001 ***
*GSTM1* Null	16	137		
*GSTT1* Present	34	83		
*GSTT1* Null	167	120	28.2	<0.001 ***

*p* < 0.001 = Highly significant (***).

**Table 2:** Genotype and allelic frequency distribution at single nucleotide polymorphism rs1695 in the *GSTP1* gene among the case and control groups enrolled in the present study, and their possible association with chronic periodontitis. The chi-square test was applied to compare the genotype and the allelic frequency between the case and control groups.

Parameters	Genotypic Frequency				*p*-Value	Allelic Frequency			*p*-Value
**Nucleotide**	**AA**	**AG**	**GG**	**Chi-Square Value**	*p* ˂ 0.0001 ***	**A**	**G**	**Chi-Square Value**	*p* ˂ 0.0001 ***
Controls	177 (88%)	20 (10%)	4 (2%)	51.348		374 (93%)	28 (7%)	65.507	
Case	115 (57%)	59 (29%)	29 (14%)			289 (71%)	117 (29%)		

*p* < 0.001 = Highly significant (***).

**Table 3:** Genotype and allelic frequency distribution among case and control subjects for combinations of GSTs and their possible association with chronic periodontitis. The chi-square test was applied to compare the genotype and allelic frequency between the case and control groups.

Genotype	Control 202	Case 203	Chi-Square	*p*-Value
*GSTM1 and GSTT1*				
Both present	34	17	48.346	*p* < 0.0001 ***
Either Null	154	116		
Both Null	14	70		
*GSTM1* and *GSTP1*(rs1695)				
M1(+/+) and P1(AA)	164	42	172.428	*p* < 0.0001 ***
M1(+/+) and P1(AG)	19	16		
M1(+/+) and P1(GG)	3	8		
M1(−/−) and P1(AA)	13	73		
M1(−/−) and P1(AG)	1	43		
M1(−/−) and P1(GG)	2	21		
*GSTT1* and *GSTP1*(rs1695)				
T1(+/+) and P1(AA)	27	47	79.023	*p* < 0.0001 ***
T1(+/+) and P1(AG)	6	27		
T1(+/+) and P1(GG)	1	8		
T1(−/−) and P1(AA)	152	68		
T1(−/−) and P1(AG)	14	32		
T1(−/−) and P1(GG)	2	21		
*GSTT1*, *GSTM1* and *GSTP1* (rs1695)				
T1 and M1(+/+) and P1 (AA)	27	12	173.564	*p* < 0.0001 ***
T1 and M1(+/+) and P1 (AG)	6	4		
T1 and M1(+/+) and P1 (GG)	1	1		
T1(+/+), M1(−/−) and P1 (AA)	1	35		
T1(+/+), M1(−/−) and P1 (AG)	1	23		
T1(+/+), M1(−/−) and P1 (GG)	1	9		
T1(−/−),M1(+/+) and P1 (AA)	135	30		
T1(−/−), M1(+/+) and P1 (AG)	13	12		
T1(−/−), M1(+/+) and P1 (GG)	2	7		
T1(−/−), M1(−/−) and P1 (AA)	13	38		
T1(−/−), M1(−/−) and P1 (AG)	1	20		
T1(−/−), M1(−/−) and P1 (GG)	1	12		

Abbreviations: *p* < 0.001 (***) = Highly significant; +/+ indicates that two copies of a specific gene are present; −/− indicates that two copies of a specific gene are absent; *GSTP1*: Glutathione S-transferase pi; *GSTM1*: Glutathione S-transferase mu; *GSTT1*: Glutathione S-transferase theta.

**Table 4:** Analysis of the studied demographic parameters and their association with chronic Periodontitis. The *p*-value indicates the results of the chi-square test calculated for each parameter.

Parameters	Category	Chronic Periodontitis Cases (N = 203)	Healthy Control (N = 201)	Chi-Square Value	*p*-Value
Age (Years)	10–30	94	02	123.692	*p* ˂ 0.001 ***
	31–51	78	110		
	52–72	29	88		
	Above 72	02	01		
Gender	Male	105	105	0.011	0.9
	Female	98	96		
Smoking	Yes	29	24	0.487	0.5
	No	174	177		

*p* > 0.05 = Non-significant, *p* < 0.001 = Highly significant (***).

## Data Availability

All the data generated during this study is presented in tables.

## Supplementary Materials

The following supporting information can be downloaded at: https://www.mdpi.com/article/10.3390/genes14020455/s1, Table S1: Genotype and allele frequencies, heterozygosity value and x^2^ value at single nucleotide polymorphism rs1695 in *GSTP1* gene among case and control groups enrolled in the present study.

## References

[1] Petersen P.E., Baehni P.C. (2012). Periodontal health and global public health. Periodontology.

[2] Armitage G.C., Cullinan M.P., Seymour G.J. (2010). Comparative biology of chronic and aggressive periodontitis: Introduction. Periodontology.

[3] Otomo-Corgel J., Pucher J.J., Rethman M.P., Reynolds M.A. (2012). State of the Science: Chronic Periodontitis and Systemic Health. J. Evid. Based Dent. Pract..

[4] Garlet G.P. (2010). Destructive and protective roles of cytokines in periodontitis: A re-appraisal from host defense and tissue destruction viewpoints. J. Dent. Res..

[5] Graetz C., Mann L., Krois J., Sälzer S., Kahl M., Springer C., Schwendicke F. (2019). Comparison of periodontitis patients’ classification in the 2018 versus 1999 classification. J. Clin. Periodontol..

[6] Kornman K.S., Papapanou P.N. (2020). Clinical application of the new classification of periodontal diseases: Ground rules, clarifications and “gray zones”. J. Periodontol..

[7] Fahim A., Shakeel S., Shahid T., Anwar H., Raja A., Khan A. (2021). Prevalence of Periodontitis in Pakistan: A Systematic Review. J. Univ. Coll. Med. Dent..

[8] Leite F.R., Nascimento G.G., Scheutz F., Lopez R. (2018). Effect of smoking on periodontitis: A systematic review and meta-regression. Am. J. Prev. Med..

[9] Shehzad S., Waheed Z., Khan K., Shah M., Durrani S.H., Farooq A. (2021). Comparison of Periodontal Diseases among Genders in Khyber Pakhtunkhwa, Pakistan. Int. J. Sci. Innov. Res..

[10] Cardoso E.M., Reis C., Manzanares-Céspedes M.C. (2018). Chronic periodontitis, inflammatory cytokines, and interrelationship with other chronic diseases. Postgrad. Med..

[11] Chapple I.L.C., Brock G., Eftimiadi C., Matthews J.B. (2002). Glutathione in gingival crevicular fluid and its relationship to local antioxidant capacity in periodontal health and disease. Mol. Pathol..

[12] Borges I., Moreira E.A.M., Filho D.W., de Oliveira T.B., da Silva M.B.S., Fröde T.S.F. (2007). Proinflammatory and Oxidative Stress Markers in Patients with Periodontal Disease. Clin. Study.

[13] Varghese J., Bhat V., Bhat G., Rao N. (2012). Evaluation of Glutathione-S-transferase and ceruloplasmin levels in gingival crevicular fluid and gingival tissue as diagnostic markers for chronic periodontitis. Adv. Biosci. Biotechnol..

[14] Nishinaka T., Ichijo Y., Ito M., Kimura M., Katsuyama M., Iwata K., Miura T., Terada T., Yabe-Nishimura C. (2007). Curcumin activates human glutathione S-transferase P1 expression through antioxidant response element. Toxicol. Letters.

[15] Sanchez F.M., Diaz A.C., Sánchez Q.H., Navarro J.B., Cadena J.C. (2019). Polymorphisms of GSTM1, GSTT1 and GSTP1 and their possible association with the development of dental caries. Pilot study. Int. J. Manag. Inf. Technol. Eng..

[16] Concolino P., Cecchetti F., D’ Autilia C., Santonocito C., Di Stasio E., Zuppi C., Arcuri C., Deli G., Giardina B., Capoluongo E. (2007). Association of periodontitis with GSTM1/GSTT1-null variants-a pilot study. Clin. Biochem..

[17] Ortega V.R.C., López L.D.B., Salgado A.V., Sanchez F.M., Cadena J.C. (2014). Polymorphisms in Glutathione S-Transferase M1, T1, and P1 in Patients with Chronic Periodontitis: A Pilot Study. Int. Sch. Res. Not..

[18] Küchler E.C., Mazzi-Chaves J.F., Antunes L.S., Kirschneck C., Baratto-Filho F., Sousa-Neto M.D. (2018). Current trends of genetics in apical periodontitis research. Braz. Oral Res..

[19] Jones S.R., Carley S., Harrison M. (2003). An introduction to power and sample size estimation. Emerg. Med. J..

[20] Jamil H., Awan A., Akbar A., Babar M., Akhtar S., Iqbal R.K., Iqbal F. (2022). A study of association between presence or absence of GSTT1 and GSTM1 and/or single nucleotide polymorphism in FABP2 and GSTP1 with incidence of diabetes type 2: A case-control study. J. Pak. Med. Assoc..

[21] Panjamurthy K., Manoharan S., Ramachandran C.R. (2005). Lipid peroxidation and antioxidants status in patients with periodontitis. Cell Mol. Biol. Lett..

[22] Hrishi T.S., Kundapur P.P., Naha A., Thomas B.S., Kamath S., Bhat G.S. (2016). Effect of adjunctive use of green tea dentifrice in periodontitis patients—A Randomized Controlled Pilot Study. Int. J. Dent. Hyg..

[23] Hernández-Ríos P., Pussinen P.J., Vernal R., Hernández M. (2017). Oxidative Stress in the Local and Systemic Events of Apical Periodontitis. Front. Physiol..

[24] Kim J.S., Park J.Y., Chung W.Y., Choi M.A., Cho K.S., Park K.K. (2004). Polymorphisms in genes coding for enzymes metabolizing smoking-derived substances and the risk of periodontitis. J. Clin. Periodontol..

[25] Jakovljevic A., Nikolic N., Carkic J., Beljic-Ivanovic K., Soldatovic I., Miletic M., Andric M., Milasin J. (2020). Association of polymorphisms in TNF-α, IL-1β, GSTM and GSTT genes with apical periodontitis: Is there a link with herpesviral infection?. Int. Endod. J..

[26] Nikita A., Andrew B., May T. (2020). Hardy-Weinberg Equilibrium in the Large Scale Genomic Sequencing Era. Front. Genet..

[27] Graffelman J., Jain D., Weir B. (2017). A genome-wide study of Hardy–Weinberg equilibrium with next generation sequence data. Hum. Genet..

[28] World Health Organization (2012). “Oral Health”, Fact Sheet no. 318. http://www.who.int/mediacentre/factsheets/fs318/en/#.

[29] Rajasekar A., Mathew M.G. (2021). Prevalence of Periodontal Disease among Individuals between 18–30 Years of Age: A Retrospective Study. Ann. Med. Health Sci. Res..

[30] Balaji S., Lavu V., Rao S. (2018). Chronic periodontitis prevalence and the inflammatory burden in a sample population from South India. Indian J. Dent. Res..

