# Supplementary material for: Association of GSTTI, M1 and Polymorphism in GSTPI with Chronic Periodontal Disease in a Pakistani Population
Source: Genes (Basel). 2023 Feb 10;14(2):455. doi: 10.3390/genes14020455 (PMC9957474; doi:10.3390/genes14020455)
Supplement: Supplementary file 1 [file genes-14-00455-s001.zip › genes-2185555-supplementary.pdf]

genes

MDPI

Supplementary Table S1. Genotype and allele frequencies, heterozygosity value, and x^2value at single nucleotide polymorphism rs1695 in GSTP1 gene among case and controls enrolled during present study. P value represents the output of Hardy-Weinberg equation calculated for cases and controls.

**Parameters**	**Genotypic frequency**			**Allelic frequency**		**X^2 value**	**P-Value**
**Nucleotide**	**AA**	**AG**	**GG**	**A**	**G**		
Controls (HO)	177 (88%)	20 (10%)	4 (2%)	374 (93%)	28 (7%)		
HE	173.9751	26.0498	0.9751	0.9303	0.0697	**10.8409**	**< 0.01**
Case (HO)	115 (57%)	59 (29%)	29 (14%)	289 (71%)	117 (29%)		
HE	101.7363	82.5274	16.7363	71.14	28.84	**17.7542**	**<0.01**

P < 0.01 = Significant. HO = Observed heterozygosity and HE = Expected heterozygosity.
